# Increased Oxidative Stress Impairs Adipose Tissue Function in Sphingomyelin Synthase 1 Null Mice

**DOI:** 10.1371/journal.pone.0061380

**Published:** 2013-04-12

**Authors:** Masato Yano, Tadashi Yamamoto, Naotaka Nishimura, Tomomi Gotoh, Ken Watanabe, Kazutaka Ikeda, Yohei Garan, Ryo Taguchi, Koichi Node, Toshiro Okazaki, Yuichi Oike

**Affiliations:** 1 Department of Molecular Genetics, Faculty of Life Sciences, Kumamoto University, Kumamoto, Japan; 2 Department of Cardiovascular Medicine, Saga University, Saga, Japan; 3 Department of Bone & Joint Disease, National Center for Geriatrics and Gerontology, Japan; 4 Department of Metabolome, Graduate School of Medicine, University of Tokyo, Tokyo, Japan and CREST, JST, Tokyo, Japan; 5 Division of Clinical Laboratory Medicine and Hematology/Oncology, Faculty of Medicine, Tottori University, Tottori, Japan; MUSC SC College of Pharmacy, United States of America

## Abstract

Sphingomyelin synthase 1 (SMS1) catalyzes the conversion of ceramide to sphingomyelin. Here, we found that SMS1 null mice showed lipodystrophic phenotype. Mutant mice showed up-regulation of plasma triglyceride concentrations accompanied by reduction of white adipose tissue (WAT) as they aged. Lipoprotein lipase (LPL) activity was severely reduced in mutant mice. *In vivo* analysis indicated that fatty acid uptake in WAT but not in liver decreased in SMS1 null compared to wild-type mice. *In vitro* analysis using cultured cell revealed that SMS1 depletion reduced fatty acid uptake. Proteins extracted from WAT of mutant mice were severely modified by oxidative stress, and up-regulation of mRNAs related to apoptosis, redox adjustment, mitochondrial stress response and mitochondrial biogenesis was observed. ATP content of WAT was reduced in SMS1 null mice. Blue native gel analysis indicated that accumulation of mitochondrial respiratory chain complexes was reduced. These results suggest that WAT of SMS1 null mice is severely damaged by oxidative stress and barely functional. Indeed, mutant mice treated with the anti-oxidant *N*-acetyl cysteine (NAC) showed partial recovery of lipodystrophic phenotypes together with normalized plasma triglyceride concentrations. Altogether, our data suggest that SMS1 is crucial to control oxidative stress in order to maintain WAT function.

## Introduction

Sphingolipids play vital roles in stabilizing membrane structure and in cell-cell recognition [1]–[5]. Some sphingolipid intermediates also act as signaling molecules in angiogenesis, cell growth, differentiation, and apoptosis [6]–[11]. One intermediate, ceramide, is a key metabolite in both anabolic and catabolic pathways, where it mediates cell differentiation, stress responses and apoptosis [6], [12], [13]. Although perturbed ceramide homeostasis reportedly accompanies many diseases [14], [15], the crucial role of ceramide in pathological states has not been fully characterized.

The initial step of sphingolipid synthesis is formation of ceramide from serine and fatty acyl-CoA in the endoplasmic reticulum (ER) [16], [17]. Synthesized ceramide is then transported to the Golgi complex by the ceramide transfer protein CERT [18], [19], where ceramide is converted to sphingomyelin by sphingomyelin synthase 1 (SMS1) [20]. Sphingomyelin is further transferred to the plasma membrane by exocytic vesicles and converted back to ceramide by sphingomyelin synthase 2 (SMS2) [17]. Thus, SMS1 has a central role in controlling sphingolipid homeostasis.

Recent genetic analysis of ceramide trafficking reveals that ceramide functions in numerous essential activities. CERT mutant mice exhibit embryonic lethality due to mitochondrial degeneration [21]. CERT null flies also exhibit a higher oxidative stress response and a shortened lifespan [22]. SMS2 mutant mice exhibit an attenuated inflammatory response in macrophages [23], decreased atherosclerosis [24] and resistance to high fat diet-induced obesity [25]. Analyses of SMS1 activity undertaken in cultured cells indicate that SMS1 has an important function in lymphoid cell proliferation [20]. Membrane sphingomyelin levels regulated by SMS1 and SMS2 activity are reportedly important for Fas translocation into lipid rafts, which promotes Fas-mediated apoptosis [26]. SMS1 suppression results in enhanced ceramide production and apoptosis after photodamage [27]. To investigate SMS1 function *in vivo*, we recently generated SMS1 knockout (SMS1-KO) mice and found that SMS1 is required to regulate generation of reactive oxygen species (ROS) and for normal mitochondrial function and insulin secretion in pancreatic β-cells [28]. In addition, SMS1-KO mice exhibited loss of epididymal white adipose tissue (WAT) mass [28]. However, the pathological consequences of that loss were not characterized.

Here, we analyzed the pathogenesis of lipodystrophic phenotypes observed in SMS1-KO mice. We report that mutant mice exhibit systemic loss of fat tissue mass. Epididymal WAT (epiWAT) mass was reduced in an age-dependent manner, accompanied by a reduction in adipose cell size. Plasma triglyceride concentrations in mutant mice increased and lipoprotein lipase (LPL) activity and fatty acid uptake activity were reduced in mutant WAT. *In vitro* analysis using cultured cells also showed reduction of fatty acid uptake by SMS1 depletion. Immunoblot analysis indicated that SMS1-KO WAT proteins were significantly modified by oxidative stress. Mutant mouse WAT showed up-regulation of mRNAs related to apoptosis, redox adjustment, mitochondrial stress response, and mitochondrial biogenesis. Reduced accumulation of mitochondrial respiratory chain complexes and lower ATP content were observed in WAT of SMS1 null mice. Treatment of mutant mice with the anti-oxidant *N*-acetyl cysteine (NAC) partially rescued these phenotypes and normalized plasma triglyceride concentrations. These data suggest that SMS1 controls oxidative stress and maintains WAT function.

## Materials and Methods

### Materials and Reagents

All reagents were purchased from Sigma-Aldrich (St. Louis, Missouri, USA) or Wako (Osaka, Japan), unless otherwise stated.

### Animal Studies

All experiments were performed using F3 generation mice. Animals were housed in a temperature-controlled room with a 12 h-light/dark cycle. Food and water were available *ad libitum* unless noted. Mice were fed a normal diet (CE-2; CLEA, Japan). NAC (40 mM) was postnatally administered in drinking water. All experimental protocols were approved by the Ethics Review Committee for Animal Experimentation of Kumamoto University.

### Metabolic Measurements

Mouse adiposity was examined by CT scanning (LaTheta; Aloka, Mitaka, Japan) as described elsewhere [29]. Plasma lipoproteins were analyzed using an HPLC system at Skylight Biotech (Akita, Japan), according to a previously described procedure [30].

### Measurement of LPL Activity

LPL activity was measured using a Total Lipase Test kit (Progen Biotechnik, Heidelberg, Germany) as previously described [31]. Briefly, tissues were homogenized in Krebs-Ringer buffer (10 mM HEPES-KOH, pH 7.4, 120 mM NaCl, 4.7 mM KCl, 2.2 mM CaCl_2_, 1.2 mM KH_2_PO_4_, 1.2 mM MgSO_4_, 5.4 mM glucose), and heparin (Ajinomoto, Tokyo, Japan) was added to a final concentration of 100 U/ml. After 45 min-incubation at 37°C, homogenates were centrifuged, and the aqueous phase was recovered and assayed. LPL activity was normalized to total protein concentration.

### In Vivo Analysis of Palmitate Uptake

Mice were deprived of food for 4 h and injected intraperitoneally with 0.02 µmol/kg [^3^H]palmitic acid bound to fatty acid-free BSA. After the indicated times, mice were sacrificed, and tissues were isolated and washed in PBS three times. Radioactivity in the tissues was measured by liquid scintillation counting and normalized to total protein concentration.

### Palmitate Incorporation Assay

Mouse embryonic fibroblasts (MEFs) isolated from wild-type and SMS1-deficient embryos were cultured in Dulbecco's modified Eagle's medium supplemented with 10% fetal calf serum at 37°C in an atmosphere of 5% CO_2_ and 95% air. For the assay, MEFs were pre-incubated in Krebs-Ringer buffer for 1 h, and then 0.05 µM [^3^H]palmitic acid bound to fatty acid-free BSA was added. After 10 min, cells were washed three times in the same buffer containing 200 µM phloretin. Cells were then lysed in water containing 0.1% SDS and the incorporated radioactive fatty acids were detected by liquid scintillation counting.

### Quantitative RT-PCR

Total RNA isolated from WAT was extracted with TRIzol reagent (Invitrogen, Carlsbad, California, USA), and DNase-treated RNA was reverse transcribed with a PrimeScript RT reagent Kit (Takara Bio, Osaka, Japan), following the manufacturer's protocol. PCR products were analyzed using a Thermal Cycler Dice Real Time system (Takara Bio), and transcript abundance was normalized to that of β-actin mRNA. PCR oligonucleotides and gene abbreviations are listed in Table S1.

### Sphingolipid Extraction and LC/ESI-MS Analysis

Total lipids in WAT were extracted by Bligh and Dyer's method [32] and analyzed using an LC/ESI-MS system composed of a quadrapole/time of flight hybrid mass spectrometer (Q-TOF micro) and an ACQUITY UPLC (Waters Corporation, Milford, Massachusetts, USA) as described previously [28], [33]. MS data processing was applied using Mass++ software (http://masspp.jp/) to detect each chromatogram peak with quantitative accuracy. The arbitrary units were respectively calculated by the peak area ratio of sphingomyelin, ceramide, or GM3 molecular species to each internal standard (sphingomyelin/d18:1-12:0, ceramide/d18:1-12:0, GM3/d18:1-14:0).

### Immunoblot Analysis

Isolated WAT or liver was homogenized in PBS containing 1% Triton X-100 supplemented with protease inhibitors. After centrifugation at 10,000× *g* for 5 min, the aqueous phase was recovered for the following immunoblot analysis. Total proteins were separated by SDS-PAGE, transferred to a nitrocellulose membrane, and analyzed using ECL Western Blotting Detection Reagents (GE Healthcare, Buckinghamshire, England) as described previously [34]. Immunoblotting was performed with anti-Hsc70 antibody (Santa Cruz Biotechnology, Santa Cruz, California, USA) or anti-4-hydroxy-2-nonenal (4-HNE) antibody (R&D Systems, Minneapolis, Minnesota, USA). When protein carbonylation was detected, total protein was separated by SDS-PAGE and transferred to PVDF membrane. The membrane was treated with 100% methanol, and then treated with TBS buffer (50 mM Tris-HCl, pH 7.4, 150 mM NaCl) containing 20% methanol. After equilibration in 2 M HCl, the membrane was incubated with 2,4-dinitrophenylhydrazone (DNPH) solution. After washing 5 times in 2 M HCl, the membrane was equilibrated in TBS buffer. The membrane was subjected to immunoblot analysis with anti-2,4-dinitrophenyl (DNP) antibody (SHIMA Laboratories, Tokyo, Japan).

### Immunohistochemical Analysis

To stain carbonylated proteins, WAT isolated from mice was fixed in a solution of 60% methanol/30% chloroform/10% acetic acid and embedded in paraffin. Specimens were randomly cut into sections. Sections were deparaffinized through 3 changes of xylene and then rehydrated through a series of graded ethanols (100%, 100%, 100%, 90%, 80%, 70%). After washing in 0.6 M HCl, sections were incubated with DNPH solution for 30 min, followed by washing in 0.6 M HCl. Sections were further washed through a series of graded alcohols (80% ethanol, 100% ethanol, 50% ethanol containing 50% ethyl acetate, 80% ethanol) and then equilibrated in water. After quenching in 1% H_2_O_2_, sections were treated with 10% normal goat serum for blocking, followed by incubation with anti-DNP antibody and secondary antibodies conjugated with horseradish peroxidase. Protein carbonylation was detected by 3,3′-diaminobenzidine (DAB) staining.

### Measurement of Caspase-3 Activity

Caspase-3 activity was measured by using caspase-3 assay kit (BioVision, Milpitas, California, USA). The assay was performed according to the manufacturer's instructions. In brief, the chromophore *p*-nitroaniline (*p*NA) after cleavage from the substrate DEVD-*p*NA was spectrophotometrically detected.

### Isolation of Mitochondria from WAT and Blue Native PAGE (BN-PAGE) Analysis

Mitochondria were isolated by the method as described previously [28], [34]. WAT were isolated and homogenized in mitochondria isolation buffer (3 mM HEPES-KOH, pH 7.5, 210 mM mannitol, 70 mM sucrose, 0.2 mM EGTA). The homogenate was centrifuged at 500× *g* to remove lipid, nuclei and unbroken cells. After removing debris through nylon filter (100 µm mesh, Clontech), the recovered aqueous phase was further centrifuged at 10,000× *g* to obtain mitochondrial pellet. The pellet was suspended in the extraction buffer containing 2% digitonin. BN-PAGE analysis was performed by the method as shown previously [35].

When immunoblot analysis was performed, proteins in the gel were transferred to PVDF membrane. After 100% methanol treatment, the membrane was washed with water, and then subjected to immunoblot analysis with anti-NDUFA9 antibody (Invitrogen), anti-ATP5A1 antibody (Invitrogen), or anti-Tom40 antibody [34].

### Measurement of Mitochondrial Respiratory Chain Activity

The gel slices obtained by BN-PAGE was used to detect mitochondrial respiratory chain activity as described [36]. Complex IV activity (cytochrome oxidase activity) was examined by incubating gel slices in the reaction buffer IV (50 mM sodium phosphate buffer (pH 7.4), 1 mg/ml DAB, 24 units/ml catalase, 1 mg/ml cytochrome *c*, 0.22 M sucrose). Color development was preserved in fixing buffer (50% methanol, 10% acetic acid), and the gel was stored in 10% acetic acid. Complex V activity (ATPase activity) was assessed by incubating gel slices in the reaction buffer V (35 mM Tris, 270 mM glycine, 14 mM MgSO_4_, 0.2% Pb(NO_3_)_2_, and 8 mM ATP). After overnight incubation, the color-developed gel was washed and stored in water. The remaining gel slice was stained with Coomassie Brilliant blue (CBB).

### Statistical Analysis

Data were analyzed using Student's *t*-test and reported as means ± SEM, unless otherwise stated.

## Results

### SMS1-KO Mice Exhibit a Lipodystrophic Phenotype

**Figure 1:**
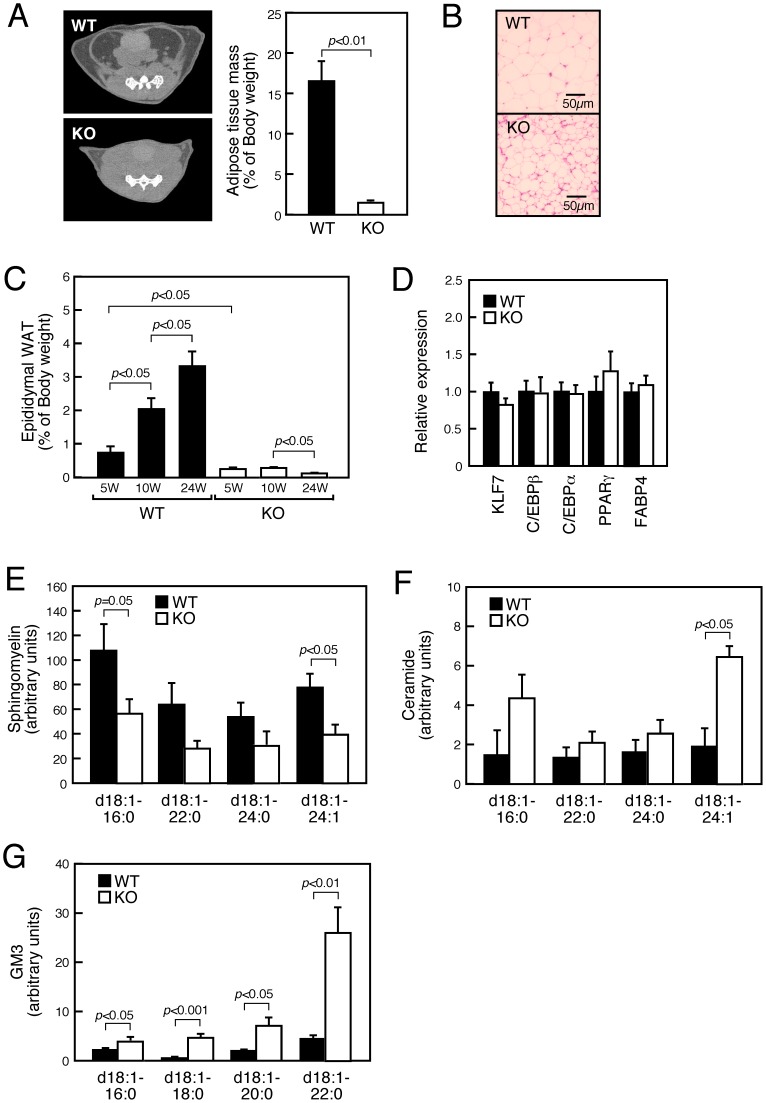
SMS1-KO mice exhibit a lipodystrophic phenotype. (A) Representative CT images of the lower abdomen (Left panel) of 20-week-old WT (*n* = 6) and KO (*n* = 4) mice and analysis of body fat mass (Right panel). (B) Sections of epiWAT from 20-week-old mice were stained with hematoxylin-eosin. (C) Age-dependent alteration of epiWAT size was observed in 5- (WT, *n* = 5; KO, *n* = 5), 10- (WT, *n* = 10; KO, *n* = 10) and 24-week-old (WT, *n* = 5; KO, *n* = 4) mice. (D) mRNA expression levels of genes encoding preadipocyte and mature adipocyte markers in WAT. Individual measurements were normalized to β–actin expression, and the wild-type group average was set to 1. *n* = 8 samples per group. (E–G) Levels of sphingomyelin (E), ceramide (F) and GM3 (G) species in isolated WAT of 10-week-old mice was analyzed by LC/ESI-MS. *n* = 6 samples per group.

Previously, we reported that SMS1-KO mice appeared lean and showed decreased epiWAT mass [28]. Here we performed CT image analysis and observed that adipose tissue mass in SMS1-KO mice was severely reduced relative to that of wild-type mice (Fig. 1A). Histochemical analysis of epiWAT revealed that the size of adipose cells of SMS1-KO mice was severely reduced relative to controls, suggestive of a lipodystrophic phenotype (Fig. 1B). Indeed, the weight of SMS1-KO epiWAT decreased with advancing age (Fig. 1C).

Because insulin is a potent adipogenic hormone [37], [38], and based on our previous finding that insulin induction by glucose is decreased in SMS1-KO mice [28], we initially asked whether adipocyte differentiation in SMS1-KO WAT was perturbed. However, we did not observe overt changes in mRNA expression of the preadipocyte markers Krüppel-like factor 7 (KLF7) and C/EBPβ or of markers of mature adipocytes (C/EBPα, PPARγ and FABP4) [39], [40] (Fig. 1D, Table S1). These observations suggest that adipocyte differentiation proceeds normally in SMS1-KO mice.

Since SMS1 catalyzes ceramide conversion to sphingomyelin, an alternative possibility is that sphingolipid homeostasis is altered in WAT of SMS1-KO mice. To test this hypothesis we examined sphingolipid composition of SMS1-KO WAT (Fig. 1E–G) by LC/ESI-MS analysis and found that levels of sphingomyelin species were reduced, while levels of ceramide and monosialodihexosylganglioside (GM3) species increased. These findings support the idea that sphingolipid metabolism is disturbed in SMS1-KO WAT.

### LPL Activity and Fatty Acid Uptake Are Reduced in WAT of SMS1-KO Mice

**Figure 2:**
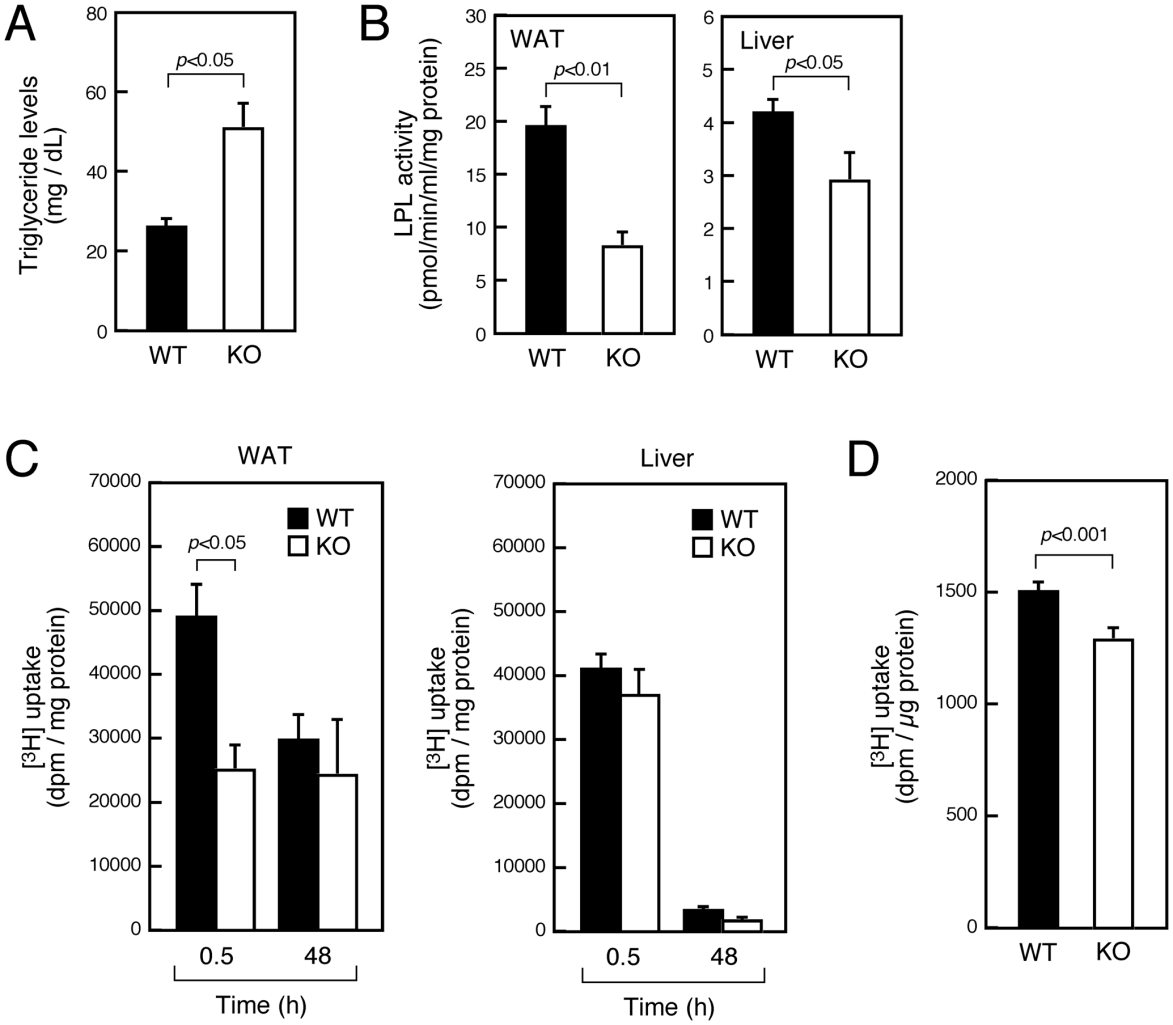
LPL activity and fatty acid uptake are reduced in WAT of SMS1-KO mice. (A) Shown are triglyceride levels in blood plasma of 14-week-old mice (WT, *n* = 3; KO, *n* = 3). (B) LPL activity was assayed in WAT and liver of 20-week-old mice (WT, *n* = 6; KO, *n* = 5). (C) [^3^H]palmitic acid was intraperitoneally injected to 4 h-starved mice. After 0.5 h (WT, *n* = 8; KO, *n* = 5) or 48 h (WT, *n* = 7; KO, *n* = 3), tissues were isolated and radioactivity in tissues was measured. (D) Assessment of [^3^H]palmitic acid uptake into MEFs isolated from wild-type (WT, *n* = 30) and SMS1-deficient (KO, *n* = 30) embryos.

We next assessed triglyceride levels in blood plasma. Those concentrations were much higher in SMS1-KO compared to wild-type mice (Fig. 2A), suggesting that metabolic functions of liver and/or WAT are perturbed.

LPL plays a critical role in triglyceride homeostasis by catalyzing hydrolysis of triglyceride from plasma lipoproteins [31]. LPL activity in both WAT and liver (Fig. 2B) was significantly reduced in SMS1-KO relative to wild-type mice, although the reduction was more severe in WAT than in liver. We next determined whether fatty acid uptake is altered in WAT and liver of SMS1-KO mice *in vivo* by assessing radioactivity levels in these tissues after intraperitoneal injection of radiolabeled palmitic acid. After 30 min, wild-type mice showed high radioactivity in both WAT and liver. By contrast, SMS1-KO mice showed relatively lower radioactivity in WAT after 30 min, although radioactivity in liver was comparable to that in wild-type mice. By 48 h, radioactivity in wild-type and SMS1-KO liver was almost completely absent, whereas a larger portion of radioactivity remained in WAT of both genotypes (Fig. 2C). We also undertook assays to evaluate fatty acid uptake *in vitro* and observed a slight but significant reduction of palmitate uptake in SMS1-deficient MEFs (Fig. 2D). Overall, these results suggest that incorporation of palmitate into WAT rather than liver of SMS1-KO mice is disturbed due to deficient fatty acid uptake function.

### SMS1-KO WAT Is Severely Damaged by Oxidative Stress

**Figure 3:**
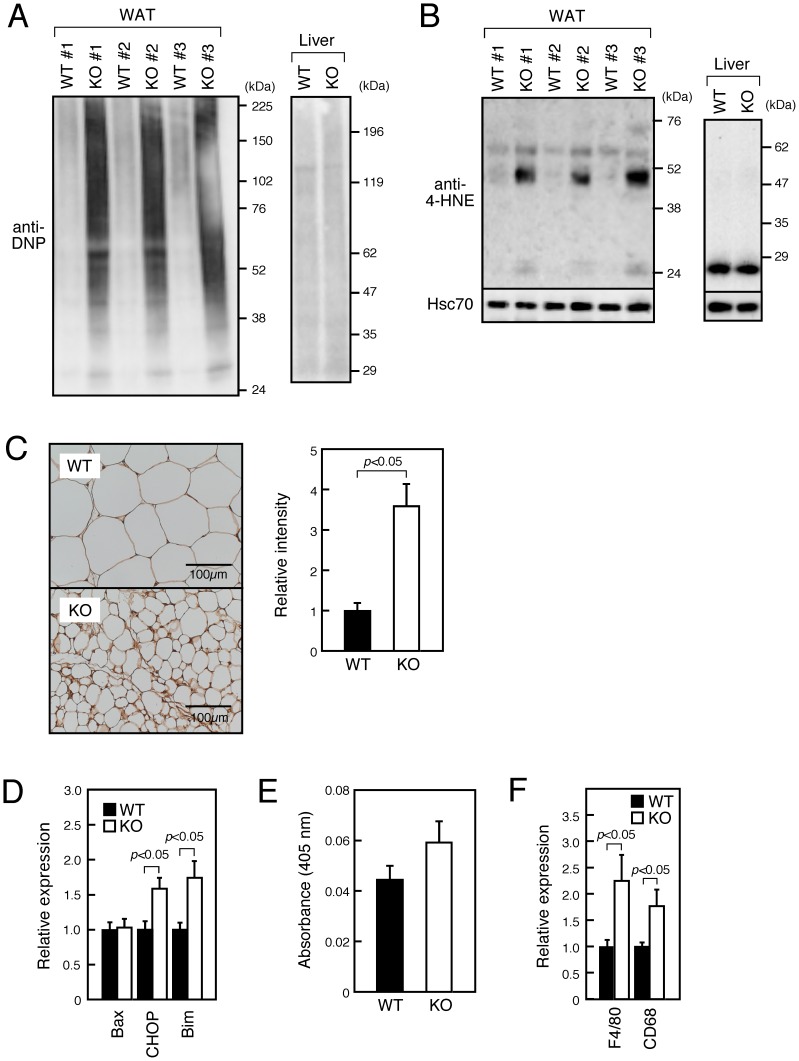
SMS1-KO WAT is severely damaged by oxidative stress. (A) Proteins were extracted from epiWAT and liver of 10-week-old wild-type (WT, *n* = 3) or SMS1-KO (KO, *n* = 3) mice and carbonylated proteins were detected by immunoblot analysis using anti-DNP antibody. (B) Samples in (A) were subjected to immunoblot analysis using anti-4-HNE antibody to detect protein modification by ROS. Hsc70 staining served as a standard. (C) Carbonylated proteins in epiWAT of 20-week-old mice were immunohistochemically detected using an anti-DNP antibody (Left panel), and DNP signal intensity in the plasma membrane area was quantified (WT, *n* = 3; KO, *n* = 3) (Right panel). (D) mRNAs were extracted from WAT of 10-week-old mice. mRNA expression levels of genes encoding apoptotic factors were assessed by quantitative RT-PCR and normalized to β–actin expression. The wild-type group average was set to 1. *n* = 6–9 samples per group. (E) Activity of caspase-3 was spectrophotometrically assessed. *n* = 6 samples per group. (F) mRNA expression levels of genes encoding macrophage-related factors were assessed by quantitative RT-PCR. *n* = 10–12 samples per group.

Previously we observed that islet cells in SMS1-KO mice were chronically damaged by oxidative stress [28]. Therefore, we asked whether oxidative stress also damaged WAT of mutant mice. Immunoblot analysis using anti-2,4-dinitrophenyl (DNP) antibody, which recognizes carbonylated proteins produced by oxidative modification [41], indicated that levels of ROS-modified proteins in SMS1-KO WAT were significantly greater than those seen in wild-type tissue (Fig. 3A). On the other hand, carbonylated proteins were not increased in the liver of SMS1-KO mice. Immunoblot analysis using the anti-4-hydroxy-2-nonenal (4-HNE) antibody, which recognizes ROS-modified proteins [42], also indicated that ROS-modified proteins were significantly increased in SMS1-KO WAT but not in the liver (Fig. 3B). Immunohistochemical analysis with an anti-DNP antibody confirmed that proteins in SMS1-KO WAT were highly modified by ROS (Fig. 3C). Interestingly, mRNA analysis showed that expression of CHOP and Bim, mRNAs encoding apoptotic factors, was increased in SMS1-KO WAT (Fig. 3D, Table S1), although we failed to observe significant increment of caspase-3 (a downstream apoptotic factor) activity (Fig. 3E). We also observed increased expression of macrophage markers (F4/80 and CD68) in SMS1-KO WAT, probably suggesting enhanced invasion of macrophage (Fig. 3F).

### Oxidative Stress Response, the Mitochondrial Stress Response and Mitochondrial Biogenesis Are Enhanced in SMS1-KO WAT

**Figure 4:**
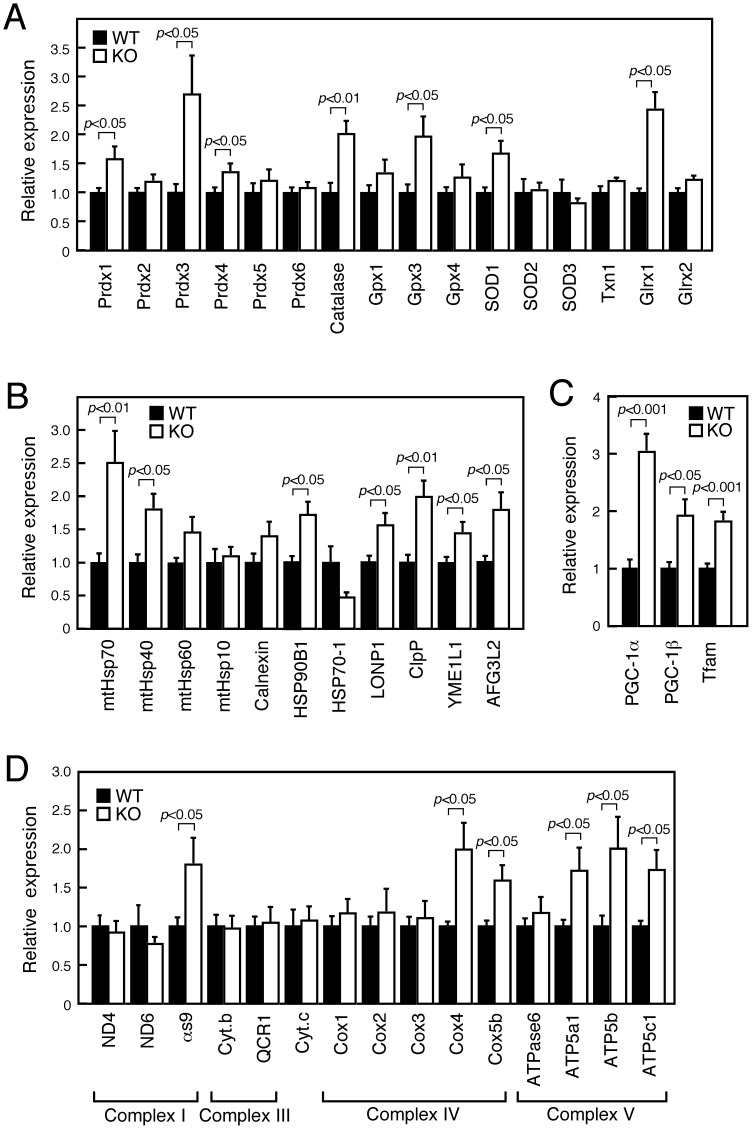
The oxidative stress response, mitochondrial stress response and mitochondrial biogenesis are activated in SMS1-KO WAT. (A–D) WAT of 10-week-old mice was analyzed for mRNA expression levels of genes encoding ROS detoxification enzymes (A), mitochondrial stress-related factors (B), mitochondrial biogenesis-related factors (C) and mitochondrial respiration complex factors (D). Values were normalized to β–actin expression. The wild-type group average was set to 1. *n* = 6–8 samples per group.

To confirm whether oxidative stress occurs in SMS1-KO WAT, we examined expression of mRNAs encoding ROS detoxification enzymes. In SMS1-KO WAT, expression of some isoforms of peroxiredoxin (Prdx), which reduces hydrogen peroxide and alkyl hydroperoxides, was increased (Fig. 4A, Table S1). Expression of catalase, which catalyzes decomposition of hydrogen peroxide, also increased. Other ROS detoxification enzymes, such as glutathione peroxidase (Gpx), superoxide dismutase (SOD) and glutaredoxin (Glrx), also showed increased expression. These data indicate that the oxidative stress response is enhanced in SMS1-KO WAT.

Next we examined expression of mRNAs encoding mitochondrial stress response-related factors. Most of these, including mitochondrial chaperones (mtHsp70, mtHsp40) and mitochondrial proteases (LONP1, ClpP, YME1L1, AFG3L2), were up-regulated in SMS1-KO WAT (Fig. 4B, Table S1). On the other hand, mRNA expression of calnexin, an ER chaperone, or HSP70-1, a cytosolic chaperone, was not affected. We also observed increases in mRNAs encoding factors relevant to mitochondrial biogenesis, such as PPARγ coactivatorsPGC-1α and PGC-1β) and mitochondrial transcription factor A (Tfam) in SMS1-KO WAT relative to wild-type tissues (Fig. 4C, Table S1). To confirm these findings, we assessed expression of components of the mitochondrial electron transport complex and ATP synthase (Fig. 4D, Table S1). Some of the former, such as αs9 (NDUFA9) and cytochrome *c* oxidase subunits IV (Cox4) and Vb (Cox5b), were up-regulated in SMS1-KO WAT relative to control tissue, as were components of the latter, such as ATP synthase, H^+^-transporting, mitochondrial F1 complex, α subunit 1 (ATP5a1), β polypeptide (ATP5b) and γ polypeptide 1 (ATP5c1).

### Mitochondrial Function Is Disrupted in SMS1-KO WAT

**Figure 5:**
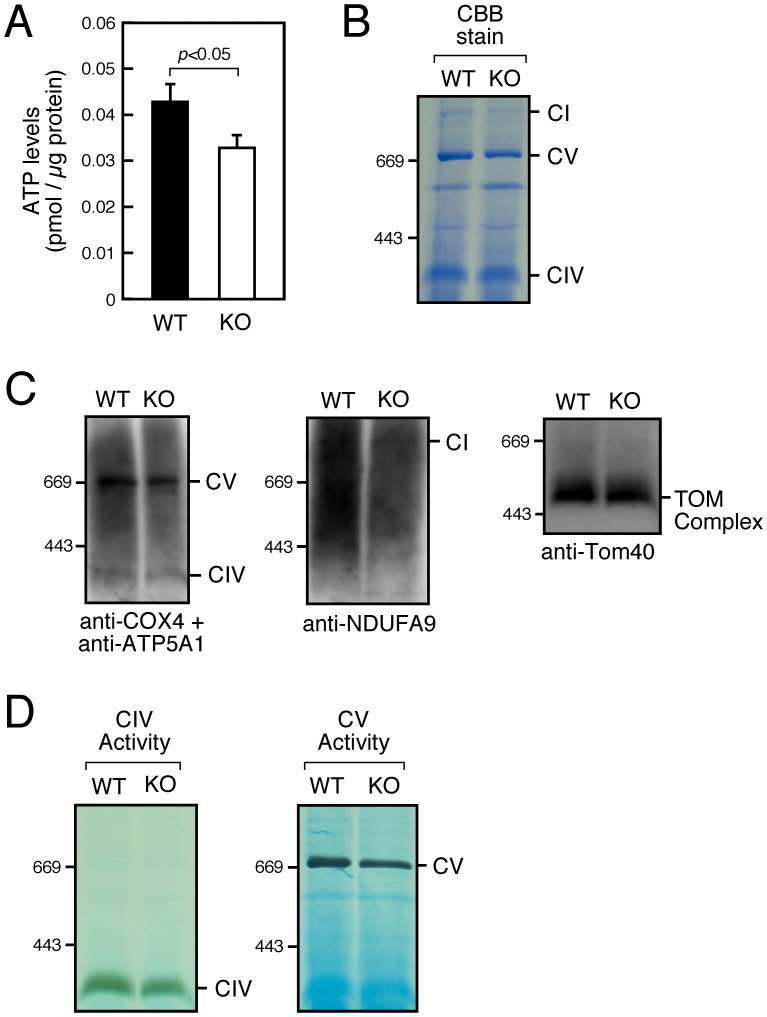
Mitochondrial function is disrupted in SMS1-KO WAT. (A) ATP content in WAT was measured (WT, *n* = 10; KO, *n* = 10). (B and C) Formation of respiratory complexes in WAT mitochondria was analyzed by BN-PAGE, followed by Coomassie blue staining (B) and immunoblot analysis (C). The complexes I, IV and V were detected by using anti-αs9 (NDUFA9), anti-COX4 and anti-ATP5A1 antibodies, respectively. TOM complex was detected by using anti-Tom40 antibody. (D) Activity of complexes IV and V was assessed (see MATERIALS AND METHODS). CI, complex I; CIV, complex IV; CV, complex V.

To examine mitochondrial function of SMS1-KO WAT, we first examined ATP level in WAT. We found that the ATP content of SMS1-KO WAT was slightly but significantly decreased compared to levels seen in wild-type mice (Fig. 5A). CBB-stain and immunoblot analysis of BN-PAGE indicated that the amounts of respiration complex I and V were reduced in the mitochondria of SMS1-KO WAT, whereas that of complex IV was not (Fig. 5B and 5C). The amount of translocase of the outer membrane (TOM complex) was not changed (Fig. 5C). These results suggest that accumulations of complex I and V are reduced in the mitochondria of SMS1-KO WAT, probably due to their instability. We further examined mitochondrial respiration complex activity in BN-PAGE gel (Fig. 5D). The activity of complex V was reduced in the mitochondria of SMS1-KO WAT, whereas that of complex IV was not. Together, these results suggest that the functions of respiration complexes in the mitochondria of SMS1-KO WAT are disturbed.

### Hyperlipidemia and Lipodystrophy in SMS1-KO Mice Are Rescued by Anti-oxidant Treatment

**Figure 6:**
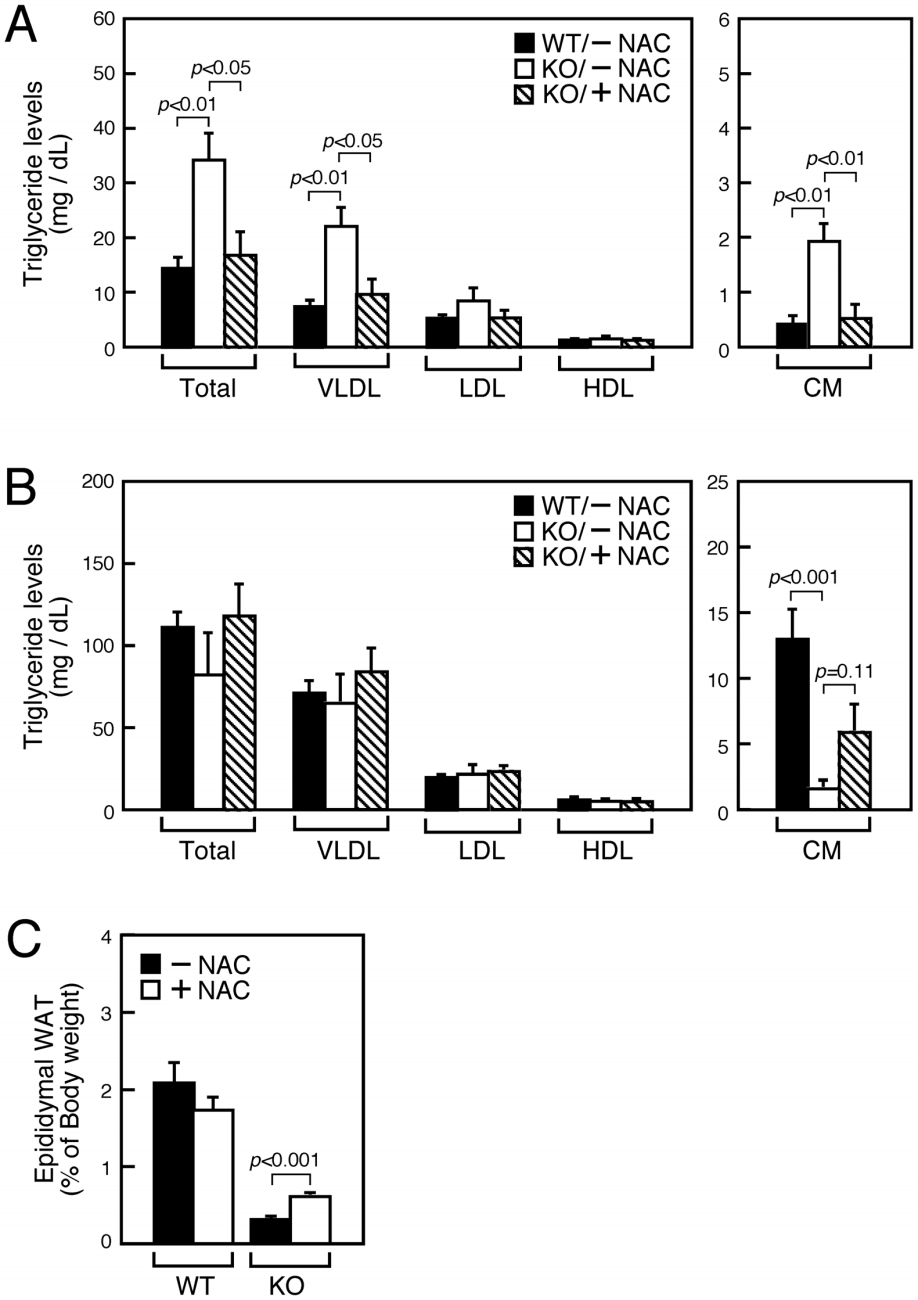
NAC treatment antagonizes WAT deficits seen in SMS1-KO mice. Mice supplied normal (−NAC) or NAC-containing (+NAC) drinking water were analyzed as follows. (A) Blood plasma isolated from 4 h-fasted mice (20-week-old) was subjected to lipoprotein analysis (WT/−NAC, *n* = 5; KO/−NAC, *n* = 4; KO/+NAC, *n* = 6). CM, chylomicron; VLDL, very-low-density lipoprotein; LDL, low-density lipoprotein; HDL, high-density lipoprotein. (B) Ten-week-old mice supplied normal (−NAC) or NAC-containing (+NAC) drinking water were subjected to 30 h-fasting. Blood plasma was collected and subjected to lipoprotein analysis. *n* = 9–10 mice per group. (C) Measurement of epiWAT size of 10-week-old mice. *n* = 10 samples per group.

Our results support the idea that WAT cells of SMS1-KO mice are functionally damaged by increased oxidative stress. Indeed, triglyceride levels were greatly increased in blood plasma of SMS1-KO compared to wild-type mice, indicative of hyperlipidemia (Fig. 6A, also see Fig. 2A). In particular, triglyceride levels of chylomicron (CM) and very-low-density lipoprotein (VLDL), which normally function in muscle or are stored in adipocytes [43], were greatly increased in blood plasma, suggesting that lipid storage function of adipose cells is compromised in SMS1-KO mice (Fig. 6A).

To further examine whether oxidative stress mediates these phenotypes, we supplied the anti-oxidant *N*-acetyl cysteine (NAC) in the drinking water of mutant mice and analyzed potential changes in blood plasma triglyceride levels (Fig. 6A). NAC-treated SMS1-KO mice showed robust decreases in blood triglyceride levels, almost to levels comparable to wild-type mice.

Finally, we subjected mice to fasting for 30 hours and assessed triglyceride levels in blood plasma (Fig. 6B). Those levels increased in fasting wild-type mice and in SMS1-KO mice supplied normal drinking water. However, CM levels in fasting SMS1-KO mice were significantly decreased compared to wild-type mice. By contrast, SMS1-KO mice supplied with NAC-containing drinking water exhibited partially recovered CM levels. The volume of SMS1-KO epiWAT was also partially rescued by NAC-treatment (Fig. 6C). Altogether, these results indicate that increased oxidative stress underlies a failure of lipid storage function in adipocytes of SMS1-KO mice.

## Discussion

Previously, we generated SMS1-KO mice and found that they exhibit phenotypes suggestive of adipose tissue dysfunction [28]. Here, we confirmed and extended those findings. Histochemical analysis revealed that the adipose cell size was severely reduced in epiWAT of SMS1-KO mice, and epiWAT volume was reduced age-dependently, suggesting that mutant mice exhibit progressive lipodystrophy. No changes were observed in expression of factors required for adipogenesis, suggesting that adipocyte differentiation proceeds normally in WAT cells of knockout mice. However, analysis of sphingolipid composition revealed reduced levels of sphingomyelin species, while ceramide and GM3 species increased in SMS1-KO WAT. Therefore, we conclude that sphingolipid metabolism is perturbed in SMS1-KO WAT cells.

Hypertriglyceridemia is reportedly a common feature of inherited lipodystrophies [44], [45]. We observed increased triglyceride levels in blood plasma of knockout mice, while LPL activity in SMS1-KO liver and WAT was reduced. *In vivo* analysis confirmed that the primary defect of SMS1-KO mice is in fatty acid uptake into WAT rather than liver. The deficiency may be a common feature among many types of SMS1-KO cells, because SMS1-deficient MEF showed a slight but significant deficiency in fatty acid uptake.

We previously showed that increased ceramide species in pancreas islets of SMS1-KO mice perturbed mitochondrial function and increased ROS generation [28]. The present study confirms that WAT of mutant mice is damaged by oxidative stress promoted by increased ROS. Others have reported that increased levels of ceramide species and oxidative stress promote cell death [46]–[48]. In agreement, we observed increased expression of apoptosis-inducing factors in SMS1-KO WAT, further evidence that the function of WAT cells is severely compromised. In addition, increased expression of macrophage markers was observed in SMS1-KO WAT, showing enhanced invasion of macrophage. These observations may indicate that damaged and dead adipocytes by oxidative stress are rapidly removed by phagocytosis by invaded macrophage in SMS1-KO WAT.

It is noteworthy that expression of ROS detoxification enzymes and mitochondrial stress response-related factors increased in SMS1-KO WAT, including components of the mitochondrial respiratory complex and factors required for mitochondrial biogenesis. These observations suggest that a mitochondrial recovery mechanism previously characterized by others [49], [50] is activated in SMS1-KO WAT. Nonetheless, we observed decreased ATP production in SMS1-KO WAT. We also observed reduced accumulations of mitochondrial respiration complexes I and V in the mitochondria of SMS1-KO WAT. The reduction of complex V activity was also observed. The reduction of these complexes is likely reflective of the profound damage brought on by oxidative stress. Indeed, the mitochondrial respiratory complex and ATP synthase are reportedly highly susceptible to modification by ROS [51]–[53]. We suppose that disorder of sphingolipid homeostasis in SMS1-KO adipocytes may cause mitochondrial protein dysfunction, that lead to increased ROS production resulting in reduced ATP production. It would be possible that these damaged adipocytes have deficiency in secretion of LPL and uptake of fatty acid.

Our conclusion that SMS1 loss interferes with lipid storage function in WAT due to oxidative stress was confirmed by the observation that levels of CM and VLDL, which are normally taken up into adipocytes [43], were increased in blood plasma of SMS1-KO mice. Anti-oxidant treatment rescued these phenotypes, indicating that reduced oxidative stress improves lipid storage function in SMS1-KO adipocytes. Analysis of SMS1-KO mice under fasting conditions further confirmed these findings. Blood CM levels in SMS1-KO mice were significantly decreased, suggesting that triglycerides derived from CM in peripheral tissues are completely consumed as an energy source. Again, anti-oxidant treatment partially normalized CM levels and also increased epiWAT volume in SMS1-KO mice. Overall, these results indicate that increased oxidative stress underlies lipid storage failure in adipocytes of SMS1-KO mice.

In conclusion, we demonstrate that manipulation of sphingolipid flux *in vivo* and consequent ceramide accumulation in WAT cells leads to oxidative stress and defects in lipid storage function. Our approach identifies an essential role for SMS1 in adipocyte function and provides molecular insight into the role of the *de novo* sphingolipid biosynthetic pathway in regulating oxidative stress, observations highly relevant to metabolic disease. To date, it has also been reported that SMS1-KO mice exhibit hearing impairment [54] and T-cell dysfunction [55]. These observations would be also attributable to loss of fundamental function of the cells by disturbance of sphingolipid. Further study is necessary to clarify the common nature of these observations.

## Supporting Information

Table S1 **Primer sequences used in quantitative RT-PCR.**
(PDF)
