# Supplementary material for: Increased Oxidative Stress Impairs Adipose Tissue Function in Sphingomyelin Synthase 1 Null Mice
Source: PLoS One. 2013 Apr 12;8(4):e61380. doi: 10.1371/journal.pone.0061380 (PMC3625169; doi:10.1371/journal.pone.0061380)
Supplement: Table S1 — Primer sequences used in quantitative RT-PCR. (PDF) [file pone.0061380.s001.pdf]

Table S1.

Target gene	Forward primer	Reverse primer
Krüppel-like factor 7 (KLF7)	CGGAGCGATGAGCTCACAAG	TCATATGGAGCGCAAGATGGTC
C/EBPβ	CTATTTCTATGAGAAAAGAGGCGTATGTAT	ATTCTCCCAAAAAAGTTTATTAAAATGTCT
C/EBPα	TCTGCGAGCACGAGACGTC	AACTCGTCGTTGAAGGCGG
Peroxisome proliferator-activated receptor-γ (PPARγ)	TTTAAAAACAAGACTACCCTTTACTGAAATT	AGAGGTCCACAGAGCTGATTCC
Fatty acid binding protein 4 (FABP4)	CACCGCAGACGACAGGAAG	GCACCTGCACCAGGGC
Bax	CAGGATGCGTCCACCAAGAA	GTTGAAGTTGCCATCAGCAAACA
CHOP	AGCTGGAAGCCTGGTATGAGGA	AGCTAGGGACGCAGGGTCAA
Bim	CGACAGTCTCAGGAGGAACC	CCTTCTCCATACCAGACGGA
Peroxiredoxin 1 (Prdx1)	ACACCCAAGAAACAAGGAGGATT	CAACGGGAAGATCGTTTATTGTTA
Prdx2	AACGCGCAAATCGGAAAGT	AGTCCTCAGCATGGTCGCTAA
Prdx3	GGCCACATGAACATCACACTGT	CAAACTGGAACGCCTTTACCA
Prdx4	TCCTGTTGCGGACCGAAT	GATCTTGGCTTTGCTTAGATGCA
Prdx5	GAAAGAAGCAGGTTGGGAGTGT	CCCAGGGACTCCAAACAAAA
Prdx6	TTGATGATAAGGGCAGGGAC	CTACCATCACGCTCTCTCCC

Catalase	GCGTCCAGTGCGCTGTAGA	TCAGGGTGGACGTCAGTGAA
Glutathione peroxidase 1 (Gpx1)	GGGACTACACCGAGATGAACGA	ACCATTCACTTCGCACTTCTCA
Gpx3	GCCAGCTACTGAGGTCTGACAGA	CCACCTGGTCGAACATACTTGAG
Gpx4	TCTGGCAGGCACCATGTGT	CGGGCATGCAGATCGACTA
Superoxide dismutase 1 (SOD1)	AGCATTCCATCATTGGCCGTA	TACTGCGCAATCCCAATCACTC
SOD2	GAGAATCTCAGTGCTCACTCGTGTC	TGCTGCCAGTCAGAAATCCAA
SOD3	CCAGCTTCGACCTAGCAGACA	CAGCGTGGCTGATGGTTGTA
Thioredoxin 1 (Txn1)	TTTCCATCTGGTTCTGCTGAGA	TGGAAGAAGGGCTTGATCATTT
Glutaredoxin 1 (Glrx1)	CAACACCAGTGCGATTCAAGA	GCAGAGCTCCAATCTGCTTCA
Glrx2	TGGAATATGGCAACCAGTTTCA	GGCGACTATCCACATCATTCAA
mtHsp70	CGTGAGCAACAGATTGTAATCCAGT	GCCATATTAACTGCTTCAACACGTTC
mtHsp40	GCCTGTATGAGACAATCAATGTGACGA	GTGAATGTAGTGGTCACCATAGCCA
mtHsp60	TCATCGGAAGCCATTGGTCATA	GCTTTGACTGCCACAACCTGAA
mtHsp10	TGCTGCCGAAACTGTAACCAA	CTTTCACACTGACAGGCTCAATCTC
Calnexin	GACTGTAGCTTTGCCAGTGTTCCT	GAGCATCCGTCTTCTTGTACTCCA

HSP90B1	ACACTTCGGTCAGGATA TCTTCTACCA	GGTTCTTCCTCCACCTG TGCTT
HSP70-1	TCTCCTGGCTGGACTCCA ACAC	ACCCTGGTACAGCCCA CTGA
LONP1	AGGAGAAGACCATTGCG GCTAA	AAAGGAGCTAAATCCG AGTAGTCCT
ClpP	TCATTGCCCAGCTGTTGT TCTTA	GGTTCAGGATGTACTG CATTGTGTC
YME1L1	AAGGCGAACTCGTCTGA TCCTC	GACAGGGTCTACCGCA GAATCAA
AFG3L2	CCACTGCGAGGATGATA GACGATG	GCTGGACCATGTCATT CTTGTCTAGG
PPARγ coactivator-1α (PGC-1α)	CCGTAAATCTGCGGGAT GATG	CAGTTTCGTTCGACCT GCGTAA
PGC-1β	GTGCCAGGTGCTGACGA GAA	AGTGTATCTGGGCCAA CGGAAG
mitochondrial transcription factor A (Tfam)	TGAAGCTTGTAAATGAG GCTTGGA	CGGATCGTTTCACACT TCGAC
Complex I, NADH dehydrogenase subunit 4 (ND4)	CATCACTCCTATTCTGCC TAGCAA	TCCTCGGGCCATGATT ATAGTAC
ND6	AGGTGAAGGCTTTAATG CTAACCC	GGTCGCAGTTGAATGC TGTGT
Complex I, α-subcomplex 9 (αs9)	CCCGGGCCAGCTTACCT	GCTGCACTGCTTTCCTG ATAGA
Complex III, Cytochrome b subunit (Cyt.b)	GCCACCTTGACCCGATT CT	TTGCTAGGGCCGCGAT AAT
Complex III, subunit core 1 (QCR1)	GCTGGGCGCACACTTTG T	CACTGGCCTTGCAGGA AGAA
Cytochrome *c* (Cyt.c)	GGCTGCTGGATTCTCTTA CACA	CCAAATACTCCATCAG GGTATCCT

Complex IV, Cytochrome Oxidase subunit 1 (Cox 1)	TTTTCAGGCTTCACCCTAGATGA	GAAGAATGTTATGTTTACTCCTACGAATATG
Cox2	CCATCCCAGGCCGACTAAA	TTTCAGAGCATTGGCCATAGAA
Cox3	CGGAAGTATTTTTCTTTGCAGGAT	CAGCAGCCTCCTAGATCATGTG
Cox4	TGCAGACCAAGCGAATGCT	TAGTCCCACTTGGCGGAGAA
Cox5b	CGTCCATCAGCAACAAGAGAATA	GCAGCCAAAACCAGATGACA
Complex V, ATP synthase 6 (ATPase6)	GGCTCCCGACACAAACTAAAAAG	TGGAATTAGTGAAATTGGAGTTCCT
ATP5a1	ATGTGTCCGCTTACATTCCAACAA	GATCCGACACGGGACACAGA
ATP5b	ACATGGGCACAATGCAGGAA	GTCAGGTCATCAGCAGGCACA
ATP5c1	AAGTTCGAAACATGGCAACTCTGA	ACTTTGCAGCTGCCACCATC
β-actin	CATCCGTAAAGACCTCTATGCCAAC	ATGGAGCCACCGATCCACA
F4/80	GAGATTGTGGAAGCATCCGAGAC	GATGACTGTACCCACATGGCTGA
CD68	CATCAGAGCCCGAGTACAGTCTACC	AATTCTGCGCCATGAATGTCC
